# Jackstone Calculus: A Spiky Cause of Haematuria

**DOI:** 10.5334/jbsr.1532

**Published:** 2018-04-19

**Authors:** Ruben Roose, Frederik Feyaerts

**Affiliations:** 1 Vrije Universiteit Brussel, BE; 2 AZ Sint-Lucas Gent, BE

**Keywords:** Jackstone, spiculated bladderstone, urinary calculus, hematuria

An 82-year-old man presented with painless, macroscopic haematuria. No other urinary complaints were reported and no previous urinary tract problems were known. Digital rectal examination revealed an enlarged prostate, confirmed on rectal ultrasound, which additionally revealed a large bladder stone and a significant amount of post-void residual urine.

CT urography was performed with the non-contrast and portal phase scanned in supine position and the delayed phase in prone position.

The prostate was enlarged and the urinary bladder showed a diverticulum at the left lateral wall.

A 2.7 cm large, spiculated and mobile calcification was seen in the urinary bladder (Figure 1, non-contrast; and Figure 2, delayed phase).

**Figure 1:**
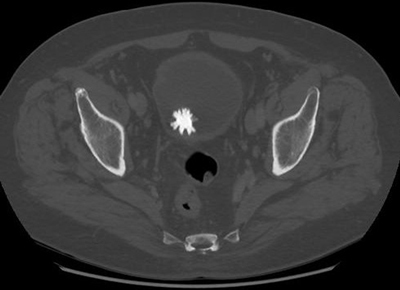
Axial non-contrast CT scan in supine position. A spiculated bladders stone, or ‘Jackstone’, is seen posteriorly in the bladder.

**Figure 2:**
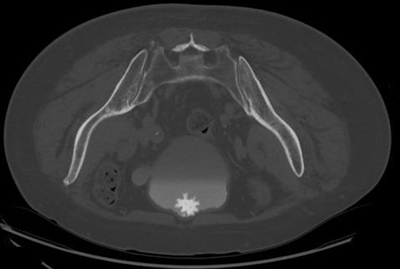
Axial delayed contrast phase CT scan in prone position. A ‘Jackstone’ is seen anteriorly in the bladder. Also notice a diverticulum at the left lateral bladder wall.

The diagnosis of a Jackstone calculus causing haematuria due to irritation of the bladder wall was made. The patient refused further treatment.

## Comment

**Figure 3:**
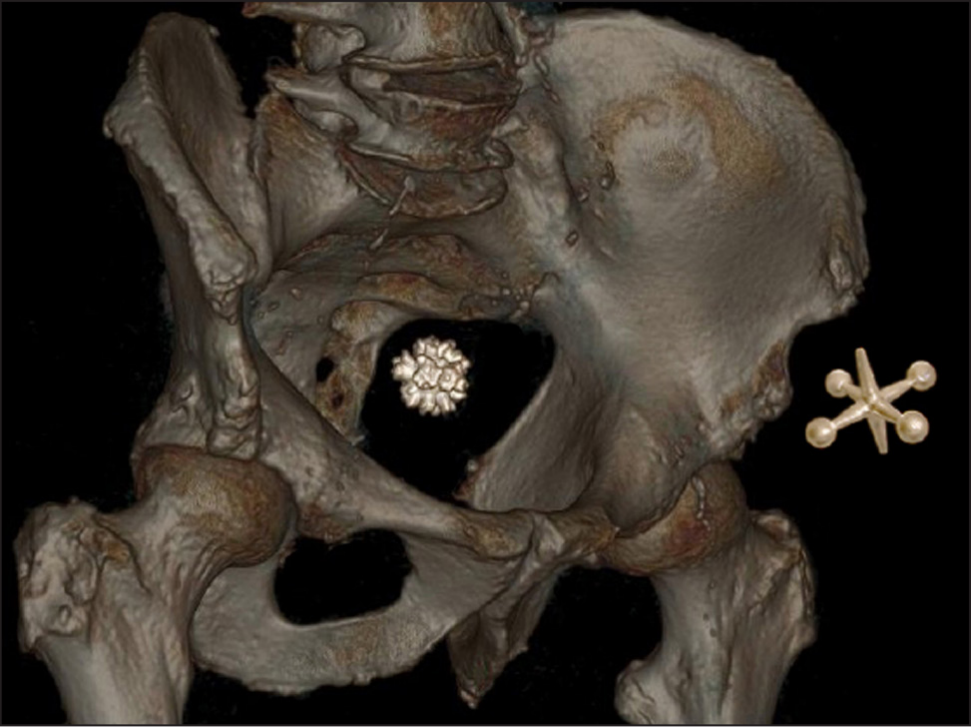
3D reformat of the non-contrast CT scan, with added real-size 3D model of a Jack toy.

A ‘Jackstone’ is a subtype of urinary calculus. It is named for its resemblance to the objects used in the children’s game ‘Jacks’ (Figure 3: 3D reformat of the non-contrast CT scan, with added real-size 3D model of a Jack toy). It has a spiculated, near-spiked appearance with a dense central core.

Urinary calculi can have different crystal compositions. This plays an important role in the crystallisation pattern and therefore, the form formation of urinary stones. Generally, calculi consist of calcium oxalate monohydrate crystals, forming dark, smooth stones. Jackstone-type calculi are usually comprised solely of calcium oxalate dihydrate crystals but can be mixed with calcium oxalate monohydrate crystals. They tend to crystallise in a specific pattern, forming spiked stones [1].

It is hypothesised that a jackstone repeatedly makes contact with the bladder wall only at the extremities of its spikes, hereby rubbing off the soft freshly precipitated apatite and any adherent mucoprotein, while allowing deposition of more calcium oxalate. Because of this, the stone grows only at the tips, producing the spiculated jackstone shape from an originally mamillated stone [1].

In our patient, prostate hypertrophy causing bladder outlet obstruction and post-void residue was the most probable cause. This is known to be an important predisposing factor to bladder stone formation.

In general, jackstones are fragmented and removed by cystoscopic or open cystolitholopaxy.
